# Identification of a novel *KCNQ1 *mutation associated with both Jervell and Lange-Nielsen and Romano-Ward forms of long QT syndrome in a Chinese family

**DOI:** 10.1186/1471-2350-9-24

**Published:** 2008-04-09

**Authors:** Su Zhang, Ke Yin, Xiang Ren, Pengyun Wang, Shirong Zhang, Lingling Cheng, Junguo Yang, Jing Yu Liu, Mugen Liu, Qing Kenneth Wang

**Affiliations:** 1 Key Laboratory of Molecular Biophysics of Ministry of Education, College of Life Science and Technology, Center for Human Genome Research, Huazhong University of Science and Technology, Wuhan, P. R. China; 2 Hubei Vocational Technical College, Xiaogan, Hubei, P. R. China; 3 Department of Cardiology, Union Hospital, Huazhong University of Science and Technology, Wuhan, P. R. China; 4 Center for Cardiovascular Genetics, Department of Molecular Cardiology, Lerner Research Institute, Cleveland Clinic, and Department of Molecular Medicine, Cleveland Clinic Lerner College of Medicine of Case Western Reserve University, Cleveland, Ohio, USA

## Abstract

**Background:**

Long QT syndrome (LQTS) is a cardiac disorder characterized by prolonged QT intervals on electrocardiograms (ECG), ventricular arrhythmias, and sudden death. Clinically, two inherited forms of LQTS have been defined: autosomal dominant LQTS or Romano-Ward syndrome (RWS) not associated with deafness and autosomal recessive LQTS or Jervell and Lange-Nielsen syndrome (JLNS) associated with deafness.

**Methods:**

A Chinese family with both RWS and JLNS was identified. Family members were diagnosed based on the presence of a prolonged QT interval as seen on a 12-lead ECG and a medical history of syncope, palpitation, and deafness. Mutational studies in the *KCNQ1 *potassium channel gene were performed using direct DNA sequence analysis and restriction length polymorphism analysis.

**Results:**

The proband in the Chinese family and her brother had previously been diagnosed with JLNS, and two other members were affected with RWS. The proband was also affected with atrial fibrillation. A single nucleotide substitution of C to T at nucleotide 965 of *KCNQ1 *was identified, and the mutation resulted in the substitution of a threonine residue at codon 322 by a methionine residue (T322M). The novel heterozygous T322M mutation was identified in two patients with RWS, one member with borderline QTc, and two normal family members. The two JLNS patients in the family carried the homozygous T322M mutation. The T322M mutation was not found in 200 Chinese normal controls.

**Conclusion:**

Our results suggest that T322M is a novel mutation that caused RWS with high intrafamilial variability in the heterozygous carriers and typical JLNS in the homozygous carriers within this Chinese family. The T322M mutation is the first mutation identified for JLNS in the Chinese population.

## Background

Long QT syndrome (LQTS) is a disorder of cardiac repolarization characterized by prolonged QT intervals and abnormal T waves on surface electrocardiograms (ECG), *torsade de pointes*, and sudden death [1-3]. Two forms of inherited LQTS have been described: Romano-Ward syndrome (RWS), which is an autosomal dominant form of LQTS without sensorineural deafness, and Jervell and Lange-Nielsen syndrome (JLNS), which is an autosomal recessive form of LQTS associated with deafness [4-6].

RWS is the most common form of inherited LQTS [7]. More than nine genes have been identified for RWS:*KCNQ1 *(or *KvLQT1*, LQT1) [8] on chromosome 11p15.5, *KCNH2 *(or *HERG*, LQT2) on chromosome 7q35-36 [9], *SCN5A *(LQT3) on chromosome 3p21 [10,11], *Ankyrin-B *(LQT4) on chromosome 4q25-27 [12], *KCNE1 *(LQT5) on chromosome 21q22 [13,14], *KCNE2 *(LQT6) on chromosome 21q22 [15], *KCNJ2 *(LQT7) 17q23.1 [16], *CACNA1C *(LQT8) on chromosome 12p13.3 [17], and *CAV3 *(LQT9) on chromosome 3p25 [18]. Carriers with mutations in *KCNJ2 *and *CACNA1C *exhibit not only the LQTS phenotype but other phenotypes as well (designated as Andersen syndrome and Timothy syndrome, respectively) [16,17].

JLNS is a rare autosomal recessive disorder that appears to have a worse prognosis than RWS [19]. JLNS can be caused by homozygous or compound heterozygous mutations in either *KCNQ1 *or *KCNE1 *[13,20-27]. *KCNQ1 *encodes a potassium channel gene with six transmembrane domains and forms functional I_Ks _potassium channels by assembling with minK (encoded by *KCNE1*) in the heart [8,28].

In this report, we identified a novel mutation in the *KCNQ1 *gene that simultaneously caused RWS and JLNS within a Chinese family. The results expand the spectrum of *KCNQ1 *mutations causing RWS and JLNS.

## Results

**Figure 1:**
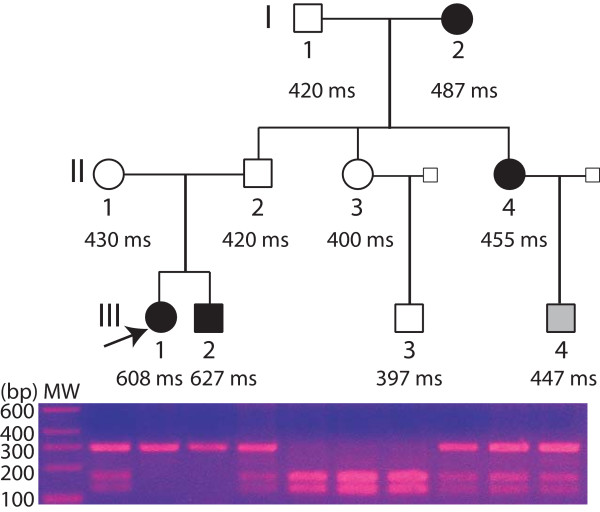
Pedigree structure of the Chinese family with JLNS and RWS. The results of RFLP analysis for mutation T322M are shown below each symbol. Affected male III:1 and females I:2 and III:2 are indicated with filled squares and circles; normal members are indicated with open symbols; individual III:4 with borderline QTc is shown with a gray symbol. QTc for family members is shown below each symbol as ms. The three-generation family is notable for the proband (III:1, indicated by an arrow) and her brother (III:2), who were affected with deafness and had a severely prolonged QTc.

**Figure 2:**
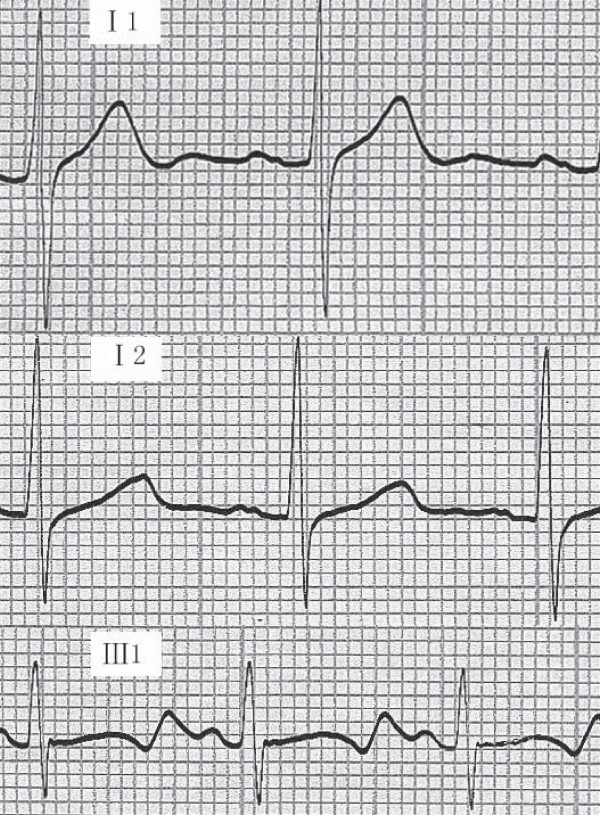
Representative electrocardiograms (ECG) from members of the Chinese family with LQTS. Top, ECG from a normal family member (I-1); Middle, ECG from a heterozygous mutation carrier; Bottom, ECG from a homozygous mutation carrier.

One three-generation JLN/RWS family was identified in China and clinically evaluated. The pedigree structure of the family is shown in Figure 1, and clinical characteristics for family members are listed in Table 1. The proband (patient III:1) had been deaf since birth. At 3 years of age, she was referred for detailed examinations due to syncope. Since then, she has experienced 11 additional syncopal episodes, most of which were preceded by exercise and sport. Current ECG analysis revealed a markedly prolonged QTc ranging from 0.520 to 0.608 s (a representative ECG is shown in Figure 2). She was then diagnosed as having JLNS (deafness + LQTS). Interestingly, the proband was also affected with atrial fibrillation. The proband's brother (patient III:2, Figure 1) was also affected with deafness and LQTS (JLNS). His QTc ranged from 0.512 s to 0.627 s, and he had experienced three syncopal episodes in the past triggered by exercise and sport. Their parents had normal hearing and normal ECGs with a QTc of 0.42 s (father) and 0.43 s (mother). Individual II:1 had experienced one syncopal episode triggered by exercise when she was 20 years old. No syncope was identified for individual II:2, but he had experienced palpitation and dyspnea. The parents' marriage was not consanguineous.

**Table 1:** Genotype-phenotype correlation in the Chinese family with both RWS and JLNS and KCNQ1 mutation T322M

**ID**	**Sex/Age**	**Deafness**	**Symptoms**	**QTc (s)**	**Genotype**
I:1	M/71	No	None	0.420	T322 T322
I:2	F/65	No	Chest discomfort	0.487	T322 M322
II:1	F/42	No	1 syncope (trigger, exercise)	0.430	T322 M322
II:2	M/43	No	Palpitation, dyspnea	0.420 0.410 0.400	T322 M322
II:3	F/39	No	None	0.400	T322 T322
II:4	F/36	No	dyspnea, palpitation	0.455 0.454 0.444	T322 M322
III:1	F/17	Yes	12 syncope (trigger, exercise) Atrial fibrillation	0.520 0.608	M322 M322
III:2	M/8	Yes	3 syncope (trigger, exercise)	0.512 0.627	M322 M322
III:3	M/16	No	None	0.397	T322 T322
III:4	M/9	No	None	0.447	T322 M322

Further analysis of other family members identified two other members affected with RWS. Individual II:4 was clinically diagnosed with LQTS because she had a moderately prolonged QTc of 0.455 s and a medical history of dyspnea and palpitation. Her mother (I:2, Figure 1) was also affected with LQTS with a prolonged QTc of 0.487 s. Both I:2 and II:4 had normal hearing. Individual III:4 was a male with a borderline QTc of 0.447 s. No stress testing was performed for III:4 or other family members. Individuals I:1, II:3, and III:3 had a normal QTc of 0.420 s, 0.400 s, and 0.397 s, respectively.

**Figure 3:**
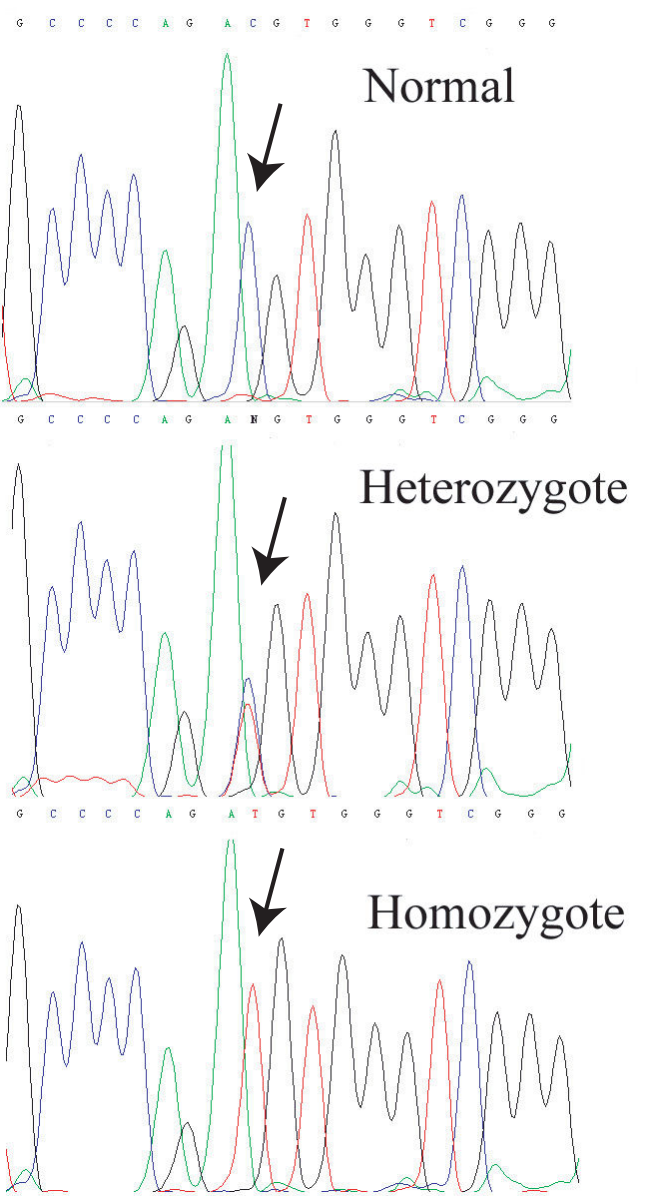
Identification of KCNQ1 mutation T322M (T to C substitution at nucleotide 965). Top, normal sequence; Middle, heterozygous T322M mutation with both T and C at nucleotide 965; Bottom, homozygous T322M mutation.

A homozygous C → T transition was identified at nucleotide 965 in exon 7 of *KCNQ1 *in the DNA sample from the proband (Figure 3). The C to T change resulted in the substitution of a threonine residue by a methionine residue at codon 322 (T322M) (Figure 3). The homozygous T322M mutation was also identified in the proband's brother (individual III:2, Figure 1). The parents and family members II:4 and I:2 were heterozygous for the mutation.

The detection of mutation T322M was further confirmed by RFLP analysis showing the presence of only the mutant allele (M322) in the proband and her brother (Figure 1, Table 1). RFLP analysis also demonstrated that family members II:1, II:2, I:2, II:4, and III:4 were heterozygous for the mutation (T322 and M322) and that normal individuals I:1, II:3, and III:3 carried the wild type T322 allele. The M322 allele was not identified in 200 normal Chinese Han nationality controls (data not shown).

## Discussion

In this report, we describe a Chinese family in which a heterozygous mutation in *KCNQ1*, T322M, resulted in RWS whereas the homozygous mutation resulted in JLNS associated with LQTS and deafness. Heterozygous mutations in the *KCNQ1 *gene have been reported in Chinese patients with RWS, but no *KCNQ1 *mutation associated with JLN has been reported in the Chinese population [29]. Thus, the T322M is the first *KCNQ1 *mutation identified for JLN in the Chinese population.

The penetrance of the phenotype in the family members with homozygous T322M was complete. The two homozygous mutation carriers, III:1 and III:2 (Figure 1), had a markedly prolonged QTc of up to 0.608 s and 0.627 s, respectively, and both had experienced multiple syncopal episodes triggered by exercise and sport. These results are consistent with the finding by Schwartz et al. [24] that JLN is a more severe variant of LQTS than RWS. Interestingly, individual III:1 was affected by both LQTS and atrial fibrillation. Previously, in a Chinese family with atrial fibrillation and KCNQ1 mutation S140G, multiple family members had both LQTS and atrial fibrillation [30]. Further studies are needed to determine whether and how the T322M mutation is associated with atrial fibrillation.

The penetrance of the phenotype of QTc prolongation in heterozygous mutation carriers was not complete. Two carriers, II-1 and II-2, had a normal QTc (Table 1, Figure 1). On the other hand, two carriers, I-2 and II:4, were affected with RWS. The other carrier, III-4, had borderline QTc prolongation. The molecular mechanism that is responsible for the intra-familial variability or variable penetrance of the LQTS phenotype in heterozygous carriers is not clear, but environmental factors and modifying genes are likely possibilities.

## Conclusion

This study identified a novel mutation, T322M, in the *KCNQ1 *gene that caused RWS with high intrafamilial variability in the heterozygous carriers and typical JLNS in the homozygous carriers within a Chinese family. To the best of our knowledge, T322M is the first mutation identified for JLNS in the Chinese population.

## Methods

### Study subjects, clinical diagnosis, and isolation of human genomic DNA

The LQTS family was identified in the Henan Province of China. Informed consent was obtained from all the participants or their guardians. This study complied with the Helsinki Declaration and was approved by the ethics committee of Huazhong University of Science and Technology.

LQTS was diagnosed based on the presence of prolonged QT intervals as seen on a 12-lead ECG and a medical history of syncope, dyspnea, and palpitation. The QT interval was manually measured, and the QTc was calculated using Bazett's formula for heart rate correction [31]. The diagnosis of LQTS was based on the work of Schwartz et al. [32] and others [8,10,11]. Asymptomatic individuals with a QTc of ≥ 0.47 s and symptomatic individuals (syncope, dyspnea, palpitation) with a QTc ≥ 0.45 s were diagnosed with LQTS. Males with a QTc of <0.44 s and females with a QTc of <0.45 s were considered normal. All others were diagnosed as having borderline QTc.

Peripheral blood was collected from the participants, and their total human genomic DNA was isolated using the DNA Isolation Kit for Mammalian Blood (Roche Diagnostic Co., Indianapolis, IN).

### Mutational analyses

Mutation screening was performed using direct DNA sequence analysis. The exons and exon-intorn boundaries of the *KCNQ1 *gene were amplified with polymerase chain reaction (PCR). PCR was performed in a 25-μL volume containing 50 mmol/L KCl, 10 mmol/L Tris-HCl, pH 8.3, 1.5 mmol/L MgCl_2_, 0.2 mmol/L of each dNTP, 0.5 μmol/L of each primer, 1 U of *Taq *polymerase (TaKaRa Biotechnology Co.), and 50 ng of genomic DNA. The amplification program consisted of one cycle of denaturation at 94°C for 3 min, 35 cycles of 94°C for 30 s, 50°C to 60°C for 30 s (with different annealing temperatures for different primer pairs), 72°C for 1 min, and one cycle of extension at 72°C for 7 min. Each PCR product was separated by a 1.5% agarose gel, purified using the QIAquick Gel Extraction Kit (Qiagen Inc., Valencia, CA) and sequenced with the forward and reverse primers using the BigDye Terminator Cycle Sequencing v3.1 kit (Applied Biosystems, Inc.).

Mutation designation was based on the cDNA sequence for KCNQ1 transcript variant 1 (Genbank accession No. NM_000218).

Mutation T322M disrupted a *MaeI *restriction site, which allowed the restriction fragment length polymorphism (RFLP) analysis to confirm the mutation in family members and to test whether the mutation was present in the controls. Using PCR, we amplified exon 7 of *KCNQ1*, which contained the T322M mutation from all members of the family as well as 200 unrelated healthy Chinese individuals of Han nationality. The PCR product was 291 bp in length and the primers were 5'-AGAGTGGTGGGTTTGGGTTAG-3' (forward primer) and 5'-GAACGTAAGTGGGTCTGCTCA-3' (reverse primer). The PCR product was digested by two units of *MaeI *at 65°C overnight and separated on a 2.5% agarose gel.

## Abbreviations

LQTS, long QT syndrome; RWS, Romano-Ward syndrome (autosomal dominant LQTS); JLNS, Jervell and Lange-Nielsen syndrome (autosomal recessive LQTS associated with deafness); ECG, electrocardiograms; PCR, polymerase chain reaction; RFLP, restriction fragment length polymorphism.

## Competing interests

The author(s) declare that they have no competing interests.

## Authors' contributions

SZ recruited the patients and family and performed the clinical and genetic studies and data analysis. KY performed genetic and clinical studies and data analysis, interpreted the results, and drafted the manuscript. XR participated in the experimental design, data analysis, interpretation of results, and supervision of the project. PW participated in genetic studies. SZ participated in genetic studies and data analysis. LC assisted with the recruitment of the patients and family and analysis of the clinical data. JY participated in the analysis of the clinical data. JYL participated in data analysis and interpretation of genetic results. ML participated in the experimental design, data analysis, interpretation of results, and supervision of the project. ML also obtained funding and helped draft the manuscript. QKW participated in the experimental design, analysis of genetic data, interpretation of the results, analysis of clinical data, and supervision of the project, obtained funding, drafted the manuscript, and critically revised the Figures, Table and entire manuscript. All authors approved the final manuscript.

## Pre-publication history

The pre-publication history for this paper can be accessed here:


